# Automated tooth segmentation as an innovative tool to assess 3D-tooth movement and root resorption in rodents

**DOI:** 10.1186/s13005-020-00254-y

**Published:** 2021-02-03

**Authors:** Viktoria Trelenberg-Stoll, Dieter Drescher, Michael Wolf, Kathrin Becker

**Affiliations:** 1 Department of Oral Surgery, Universitätsklinikum Düsseldorf, Düsseldorf, Germany; 2 Department of Orthodontics, Universitätsklinikum Düsseldorf, Düsseldorf, Germany; 3 Department of Orthodontics, Universitätsklinikum RWTH Aachen, Aachen, Germany; 4 Department of Oral Surgery and Implantology, Goethe University, Frankfurt am Main, Germany

**Keywords:** Tooth segmentation, Micro computed tomography, Animal experiment, Orthodontic tooth movement

## Abstract

**Background:**

Orthodontic root resorptions are frequently investigated in small animals, and micro-computed tomography (μCT) enables volumetric comparison. Despite, due to overlapping histograms from dentine and bone, accurate quantification of root resorption is challenging. The present study aims at (i) validating a novel automated approach for tooth segmentation (ATS), (ii) to indicate that matching of contralateral teeth is eligible to assess orthodontic tooth movement (OTM) and root resorption (RR), (iii) and to apply the novel approach in an animal trial performing orthodontic tooth movement.

**Methods:**

The oral apparatus of three female mice were scanned with a μCT. The first molars of each jaw and animal were segmented using ATS (test) and manually (control), and contralateral volumes were compared. Agreement in root volumes and time efficiency were assessed for method validation. In another *n* = 14 animals, the left first upper molar was protracted for 11 days at 0.5 N, whereas the contralateral molar served as control. Following ATS, OTM and RR were estimated.

**Results:**

ATS was significantly more time efficient compared to the manual approach (81% faster, *P* < 0.01), accurate (volume differences: − 0.01 ± 0.04 mm^3^), and contralateral roots had comparable volumes. Protracted molars had significantly lower root volumes (*P* = 0.03), whereas the amount of OTM failed to reveal linear association with RR (*P* > 0.05).

**Conclusions:**

Within the limits of the study, it was demonstrated that the combination of ATS and registration of contralateral jaws enables measurements of OTS and associated RR in μCT scans.

## Background

Root resorptions (RR) are an undesired side effect of orthodontic treatment, and apical shortening of more than 3 mm was estimated to affect 30–35% of the patients. [1, 2].

The current biological concepts of RR have been mainly derived from small animal studies and end-point histology. [3–6]. Major drawbacks of this approach, however, are the high information loss during histological processing and the limitation to two dimensions. [7].

Micro computed tomography (μCT) is a complementary tool enabling high-resolution volumetric analyses of bone and tooth micro-morphometry. [8–14] In principle, the 3D-orthodontic tooth movement (OTM) can be computed even from end-point analyses, and they can also be correlated with the associated local hard tissue changes. However, at the time being, the majority of studies performed 2D linear measurements in the volumetric data sets, comparable to 2D histology, or they mechanically separated murine teeth from bone prior to scanning. [15].

3D evaluation premises reliable image segmentation which can be particularly challenging when metal artifacts are present, or when histograms overlap. [16–20] Since mineral contents of cementum and bone were reported to be likewise [21], reliable approaches to segment tooth roots in μCT are an inevitable premise for volumetric assessment of OTM and RR in μCT.

Marker-based Watershed algorithms (WS) have been successfully used to segment computed tomography images with overlapping histograms. [22, 23] Hence, this approach may also be applicable in the orthodontic field.

The present study aims at (1) assessing the eligibility of a WS algorithm for 3D automated tooth segmentation (ATS), (2) evaluating whether symmetry is sufficient to use contralateral hemi-maxillae to compute OTM and RR in mice, and (3) to apply the novel methodology to μCT scans from a previous animal study as prove of concept.

## Methods

### Animals

Two separate sets of specimens (mice) were used for the present investigation.

**Fig. 1:**
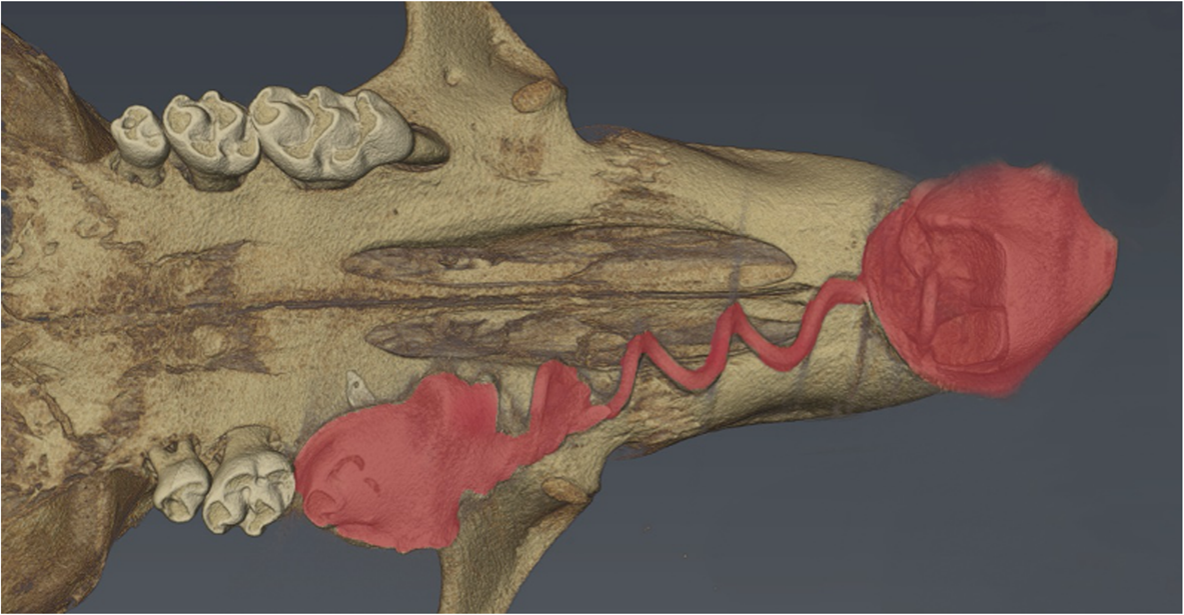
Volumetric rendering of the upper jaw from one animal. The image shows μCT scans from an animal experiment analyzing the effect of orthodontic tooth movement in mice with a nickel titanium spring (red) installed between the left upper first molar and the incisor to induce anterior movement of the first molar. Orthodontic tooth movement was conducted at 0.5 N for 11 days

For the method part, i.e. aim (i) and (ii) specified in the background, skulls from three female mice (BALB/c strain, age 5.2–5.6 month) were obtained for μCT scanning from the local animal facility of the University Hospital of Düsseldorf. For the application part, i.e. aim (iii), μCT scans from a previously described and published animal experiment analyzing the effect of orthodontic tooth movement in mice were used [24]. For that purpose, a nickel titanium spring was attached between the left upper first molar and the incisor, and molar protraction was conducted at 0.5 N for 11 days (Fig. 1). According to the study protocol the included animals had an age 60 days (9 females, 5 males) and different genetic profiles, which were out of the scope for the present investigation. A total of 14 μCT scans were available for the present investigation. All experiments were done in accordance with the appropriate animal care committees and laws (Central institution for animal research and scientific research protection tasks, University hospital of Düsseldorf, Germany; National Institute of Athritis and Musculoskeletal and Ski Diseases (NIAMS) Animal Care and Use Committee, reference number: A016–12-09).

### Micro computed tomographic analysis

For the method part, the samples were scanned with a μCT (Viva CT 80; Scanco Medical AG, Brüttisellen, Switzerland) operated at 70 kVp, 114 μA, 8-W, 31.9 mm FOV, 1500 projections, and an integration time of 500 ms. The data sets were reconstructed into three-dimensional (3D) volumes with an isotropic nominal resolution of 10.4 μm voxel size.

For the application part, the samples were scanned with a μCT 50 (Viva CT 80; Scanco Medical AG, Brüttisellen, Switzerland) operated at 70 kVp, 76 μA, 300–900 ms integration time and 9-to-10 μm voxel size.

### Image processing

Image processing was performed using Amira software (v6.5, FEI Visualization Science Group, Burlington, MA, USA) by a trained investigator (VTS) and validated by another author (KB).

#### Automated tooth segmentation

**Fig. 2:**
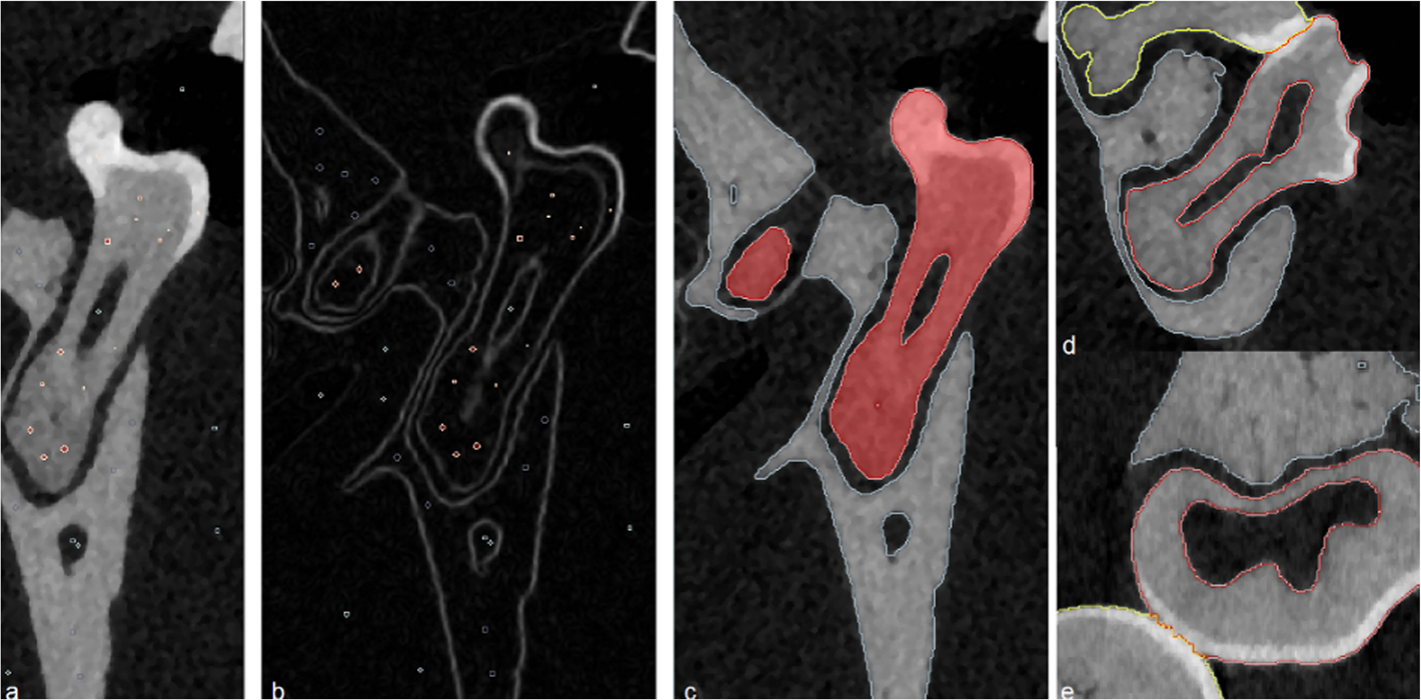
The image shows how the segmentation procedure of tooth, bone and roots were performed. **a** First, the image was prepared by marking the structures to be identified with different colors (labels). **b** Second, the edges of the structures were filtered (Sobel operator). **c** Third, each label was grown until reaching an edge (Watershed algorithm). (D/E) If no clear edge was found (e.g. at contact points between teeth), manual correction was performed. Different labels were used for “molar”, “bone”, “nickel titanium spring” and “air”

Segmentation of teeth and their roots was achieved in three steps (Fig. 2). First, the image was prepared by marking the structures to be identified with different colors (labels). Second, the edges of the structures were filtered (Sobel operator). Third, each label was grown until reaching an edge (Watershed algorithm). If no clear edge was found (e.g. at contact points between teeth), manual correction was performed. Different labels were used for “molar”, “bone”, “nickel titanium spring” and “air”.

#### Adjustment of the teeth to a uniform coordinate system

The segmented molars were aligned such that the cementum-enamel junction (CEJ) coincided with the occlusion plane (Euclidean XY-plane), and that the line connecting the mesial and distal contact points formed a 45-degree angle with the X- and Y- axis, respectively.

#### Measurement of the root volumes (mm^3^)

To calculate the volume of the tooth roots, the roots were digitally separated from the teeth. This was achieved by adding a cutting plane 30 voxels (approx. 300 μm) below CEJ.

#### Method validation

To assess eligibility and reliability of the ATS method, the manual approach (MA) served as reference. Therefore, each tooth was segmented one more time by labelling it slice-wise in the μCT scans. The roots were again separated as described above.

Both methods, i.e. ATS and MA, were repeated three times, and the respective time requirements were recorded.

#### Comparison of root volumes between hemi-maxillae

In end point analyses with split-mouth design, values from contralateral sites are compared. This premises comparability. To validate this assumption, the volumes of roots from contralateral molars were compared (see statistics section).

#### Measurement of orthodontic tooth movement and root resorption (application)

**Fig. 3:**
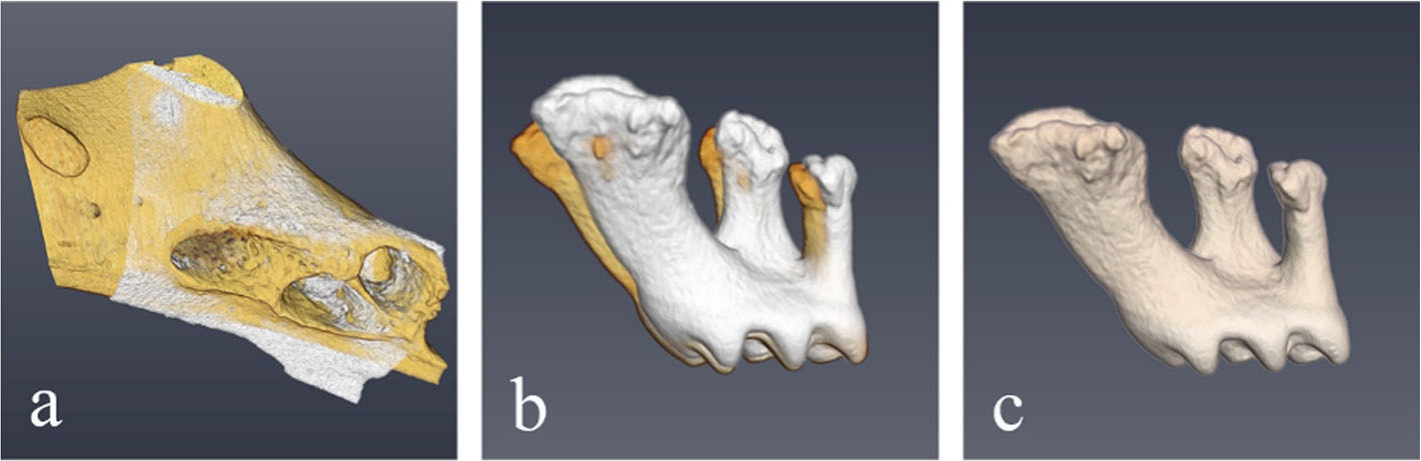
Superimposition process used to analyze tooth movement of upper first molar with the contralateral molar as internal reference. **a** First the control site had to be transformed using a mirror operation. **b** Second, the transformed image was superimposed using the bone as reference (only the registered teeth are shown) (Fig. 3b). **c** Then, OTM was quantified using the reference tooth as control reference structure for another image registration (due to high symmetry, the contralateral roots are overlapping, the figure also represents the symmetry of contralateral teeth)

To assess OTM, the contralateral site was used as reference. Since this site is mirror-symmetric, it had to be transformed accordingly (mirror-operation, Fig. 3a). After this, image registration was performed using the bone tissue as reference (Fig. 3b). Then, OTM was computed by using the unprotracted control-molar as reference structure (Fig. 3c). To estimate RR, the respective root volumes from the test and control site were subtracted to assess the absolute hard tissue loss, whereas their fraction was computed to assess the relative hard tissue loss.

### Statistical analysis

The statistical analysis was performed using the software program R. [25].

For descriptive purposes, medians and quartile ranges were computed for each variable and group, and either represented in text or in boxplots.

To perform method validation, Bland-Altman analyses were employed to assess agreement between ATS and MA, and the three repeated measurements are represented by equal color and symbol in the respective Bland-Altman plots. Reliability of the segmentation procedures was analyzed by computing the respective intra class correlation coefficients (ICC). The time needed for segmentation (efficiency) was compared using the Wilcoxon signed rank test.

The Wilcoxon signed rank test was also used to compare contralateral root volumes.

The paired t-test was used to compare OTM and RR following tooth protraction (normal distribution was validated in advance). Linear regression was employed to assess linear association between tooth movement and root resorption (variance homogeneity and normal distribution of residuals were validated in advance). Results were found significant at *P* < 0.05.

## Results

### Eligibility of ATS for automated tooth segmentation

Automated tooth segmentation was successfully achieved in all animals. For unprotracted teeth, manual correction had to be used in the interproximal contact areas.

**Fig. 4:**
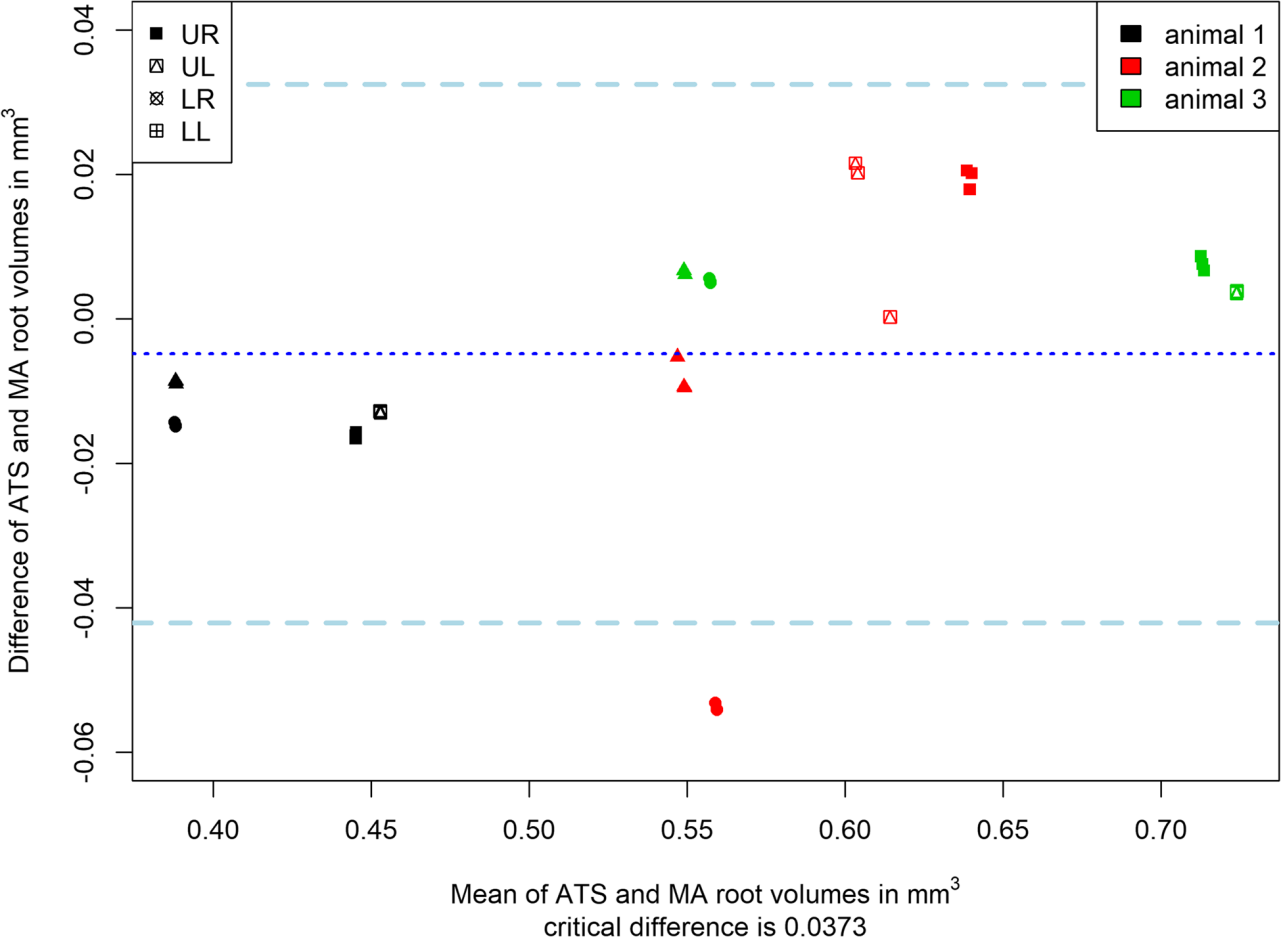
Bland-Altman analysis was performed to compare root volumes between the two segmentation approaches (ATS vs. MA). Measurements from the same animal are labelled with equal colour, and the position of the teeth (upper right UR, upper left UL, lower left LL, lower right LR) is labeled by icons. Median tooth segmentation time amounted to 8.93 min (quartiles: 8.13–11.76 min) for ATS, and to 55.26 min (quartiles: 49.62–62.76 min) for MA. Tooth segmentation was by 81% significantly faster using ATS (*P* < 0.01)

When comparing the tooth and root volumes with the respective reference value from MA segmentation, the Bland-Altman analyses revealed a mean difference of 0.01 mm^3^ (critical difference: 0.04 mm^3^) for tooth roots and 0.03 mm^3^ (critical difference: 0.06 mm^3^) for the teeth (Fig. 4). Reliability of repeated measurements was high (ATS: ICC = 1.00, MA: ICC = 0.998).

Median segmentation time amounted to 8.93 min (quartiles: 8.13–11.76 min) for ATS, and to 55.26 min (quartiles: 49.62–62.76 min) for MA. Tooth segmentation was 81% faster using ATS (*P* < 0.01).

### Volume comparison for contralateral molars

The total volumes of the left and right first molars amounted to 0.548 ± 0.117 mm^3^ and 0.543 ± 0.114 mm^3^, respectively (ATS segmentation). Comparison of contralateral sites did not reveal any significant difference (ATS segmentation: *P* = 1.0, MA segmentation: *P*=0.41).

In addition, visual examination by flipping the right molar along the sagittal plane and subsequent registration with the contralateral tooth confirmed symmetry (the same approach was used to assess tooth movement in the application part, see Fig. 3).

### OTM and associated RR

**Fig. 5:**
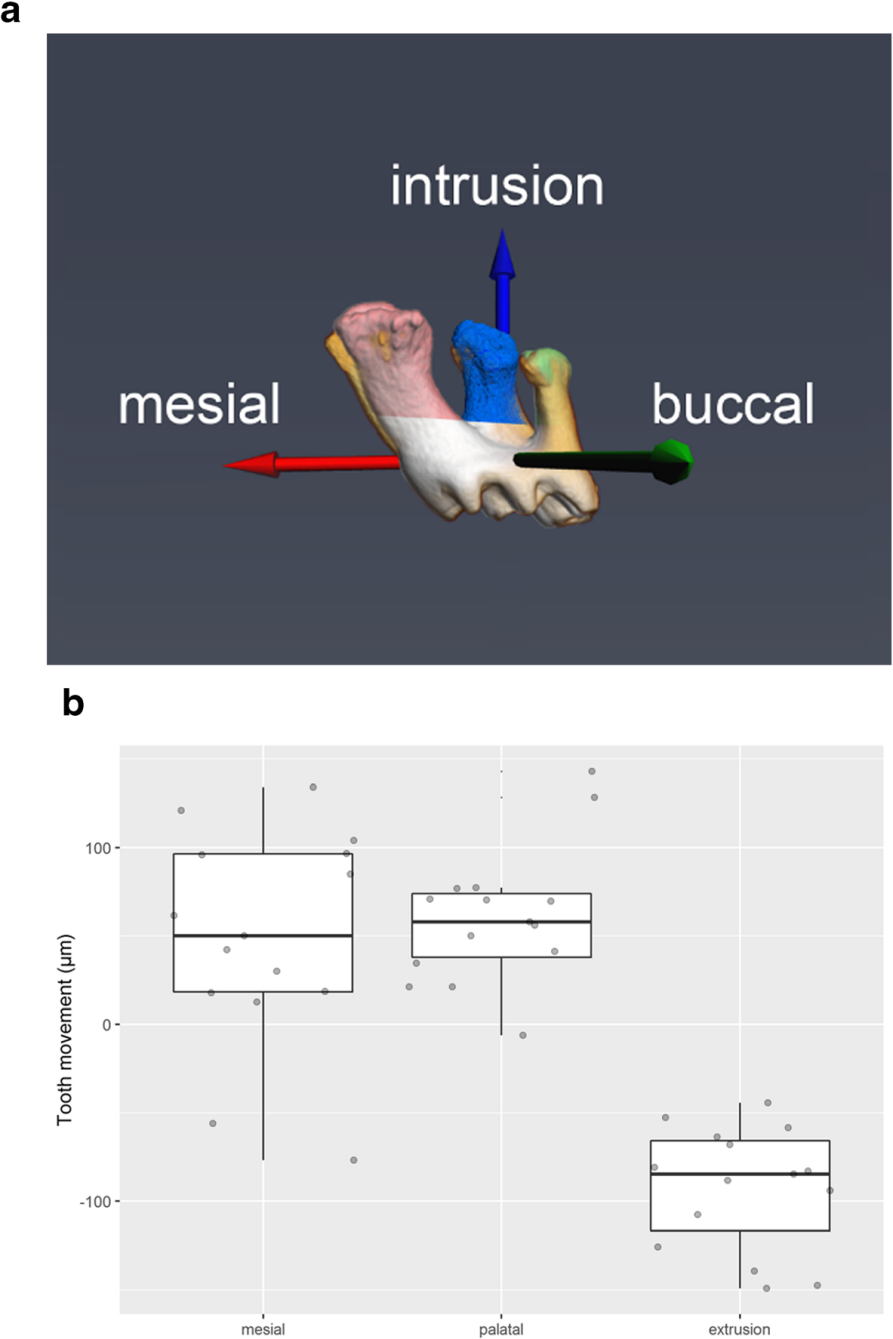
**a** Schematic drawing representing the mean amount of orthodontic tooth movement in the mesial, buccal and vertical dimension (intrusion: 0.08 mm, mesial displacement: 0.04 mm, palatal displacement: 0.06 mm). **b** Boxplot representing the amount of experimental orthodontic tooth movement of the upper molar in all 3 dimensions. A significant amount of tooth movement in mesial direction associated with palatal rotation and intrusion could be observed

The mean tooth movements in all 3 dimensions mainly occurred to the mesial, palatal and intrusion direction. Following the 3D analyses the analyzed tooth movement could be interpreted as an anterior movement with mesial and palatal tipping and molar intrusion (intrusion: 0.08 mm, mesial: 0.04 mm, palatal: 0.06 mm) (Fig. 5).

**Fig. 6:**
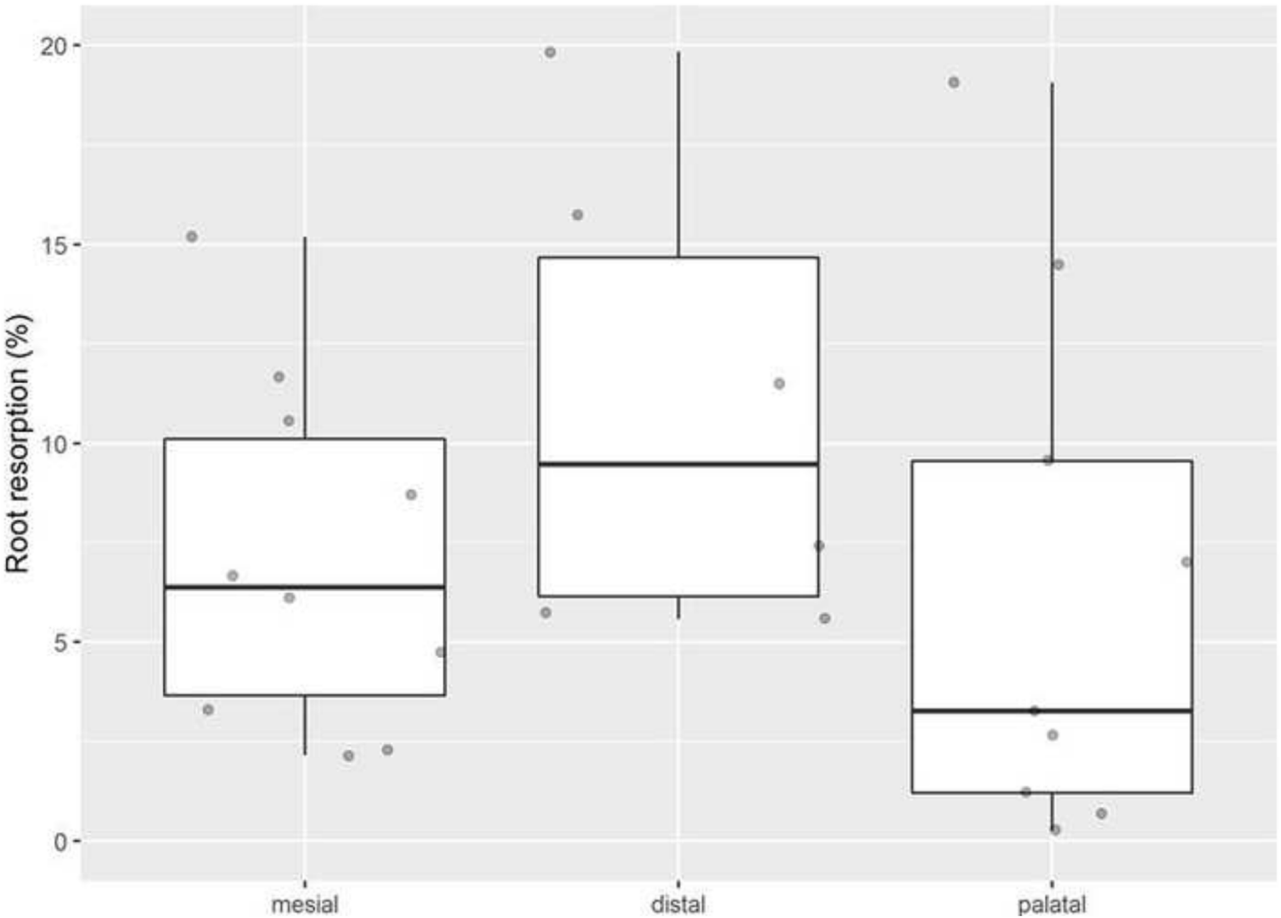
Effect of tooth movement on tooth root resorption (%). The graph shows the relative volumetric differences for each root in relation to the non-treated reference site

Comparison of root volumes of upper first molars after 11 days of experimental tooth movement revealed a minor but significant loss of hard tissue at the mesial (control: 2,085,540, Q1-Q3 1,904,077-2,555,135 μm^3^ vs. test: 1,986,680, Q1-Q3: 1,717,100-2,411,930 μm^3^ (*P* = 0.003)), distal (control: 775,220, Q1-Q3: 646,835–973,610 μm^3^ vs. test: 659,925, Q1-Q3: 617,975–894,055 μm^3^ (*P* = 0.012)), and palatal root (control 1,139,960, Q1-Q3: 881,375-1,454,115 μm^3^ vs. test 1,109,430, Q1-Q3: 868,145-1,299,900 μm^3^ (*P*=0.033)) (Fig. 6). Interestingly, the absolute loss of hard tissue was highest in the mesial root, whereas relative reduction **(%)** of root volume compared to the none treated control site was comparable in the mesial and distal root (mesial: 6.08 ± 5.85%, distal: 5.66 ± 7.78%, palatal: 3.65 ± 6.75%).

Linear regression analysis did not reveal association between OTM and root resorption (*P* = 0.69, R^2^ = − 0.06).

## Discussion

Micro-CT is frequently used for evaluation of hard-tissue biopsies. Whereas open access software is available to assess bone micro-structural parameters [26], calculation of orthodontic tooth movements and volumetric assessment of hard tissue loss has been rarely investigated. In the orthodontic field, 2D-measurements between contact points are most commonly assessed to quantify tooth movements in μCT, even though they do not reflect the true 3D movement. This may own to the fact that segmentation of teeth in μCT scans is challenging because grey values from dentine and bone hardly differ. [21].

Therefore, the present study aimed at assessing whether a Watershed Algorithm is efficient for tooth segmentation, whether a split-mouth design is applicable to estimate the absolute and relative hard tissue loss following tooth protraction, and to apply the novel approach to μCT scans obtained from a previous animal study to compute orthodontic tooth movement (OTM) and root resorption (RR). [24].

In each animal, automated tooth segmentation was successfully achieved. Accuracy of the method was confirmed by the high agreement of tooth- and root volumes assessed with ATS and MA. It has to be noted that slice-wise manual segmentation was considered to be the most accurate reference. (27, 28) Careful extraction of the molars prior to (repeated) μCT scanning has also been proposed in literature [29, 30], but due to potential detrimental effects this approach was considered to be not sufficiently reliable.

When comparing the time requirement of the automated approach with the manual reference, ATS was about 81% faster. Giving the high number of biopsies that is frequently analyzed in small animal studies, this appears to be a relevant benefit of the presented approach. At this point, it has to be noted that the term *automated* specifies “procedures operated by machines or human to reduce the work done by humans” [31]. Hence, automatic and/or fully automated approaches may further increase efficiency in the future.

Split mouth designs have been frequently employed in orthodontics. [32–34] They rely on the assumption that high agreements exist between the left and right site. However, this assumption has been rarely validated, even though comparability was suggested. [35–39] For this purpose, the tooth and root volumes were compared in the untreated animals. Intra-animal agreement between contralateral sites was high, whereas marked differences were found among the animals (Fig. 4). This indicates that calculation of the relative hard tissue loss (volume of the test root divided by the volume of the control root) may be more accurate then comparing absolute values between groups. It has to be emphasized that this volumetric computation of RR is not possible from 2D images, e.g. projection radiographs or histology.

Assessing orthodontic tooth movements using the other maxilla as reference required a series of steps. First, the contralateral site had to be reversed (as it is mirror symmetric) and registered with the test site. Adjustment to an intraoral coordinate system was also required to measure OTM in all three dimensions, as reported previously [29, 40]. In contrast, the subsequent second molar would enable only linear distance measurements in the interproximal area. To what extent second molars remain stationary while the first molar is protracted remains to be investigated.

In the present study, measurements of RR revealed that all roots were affected by volume loss (Fig. 6). Whereas the highest amount (mm^3^) of hard tissue loss occurred at the mesial, the greatest relative volume loss was observed at the distal root. Even though association of RR and the tooth movement has been postulated [41], no significant association was found in the present study. This is in line with a recent systematic review on clinical trials, which also failed to reveal association of RR and tooth movement. [42].

Some limitations are associated with the present investigation. In the method part, only few animals were used. Assessing OTM by means of the opposite hemi-maxilla requires symmetry, which may not always be perfect. Longitudinal in vivo μCT would overcome this limitation. Since scanning time has to be shorter for living animals scanned with an in vivo μCT, the images would be more affected by metal artifacts from the nickel titanium spring.

In the future, automatic tooth segmentation using artificial intelligence (AI) might become a fast and accurate alternative. At the time being and according to the knowledge of the authors, recent reports mainly focused on the detection (using bounding boxes) or automatic segmentation of teeth on 2D-panoramic x-rays. [43, 44] For volumetric data, very few reports exist as they require reliable 3D training data. It has to be noted that creation of training data including accurate 3D-segmentation of a huge number of data sets can be extremely time consuming. [45] Therefore, the present approach may also be used for efficient creation of training data for AI based segmentation using convolutional neural networks in future studies.

## Conclusions

The present study demonstrated that the Watershed Algorithm is applicable for tooth root segmentation in μCT images. Comparable root volumes and shapes were found between contralateral teeth, suggesting that estimating root resorption in split-mouth studies is eligible. Application to an animal experiment analyzing molar protraction revealed that the contralateral site may also serve as reference to compute OTM in end-point analyses.

## Data Availability

Due to the size, the Micro CT- Scans will be available upon request to the authors. All other data is included in the publication.
